# Asymptomatic carriage of *Streptococcus pyogenes* among school children in Sana’a city, Yemen

**DOI:** 10.1186/s13104-019-4370-5

**Published:** 2019-06-14

**Authors:** Arwa Mohammed Othman, Rowa Mohammed Assayaghi, Huda Zaid Al-Shami, Riyadh Saif-Ali

**Affiliations:** 1 Microbiology and Immunology Department, Faculty of Medicine and Health Sciences, Sana’a University, Sanaa, Yemen; 2 Biochemistry Department, Faculty of Medicine and Health Sciences, Sanaa University, Sanaa, Yemen

**Keywords:** Asymptomatic carriers, Pharyngeal swab, School children, *Streptococcus pyogenes*, Yemen

## Abstract

**Objectives:**

*Streptococcus pyogenes* is the most frequent cause of pharyngitis and skin infections in children. It is also the causative agent of dangerous immune-complications such as rheumatic fever and rheumatic heart disease which are common in Yemen. The aim of this study was to determine the throat carriage rate of *Streptococcus pyogenes* among asymptomatic school children in Sana’a city.

**Results:**

A cross-sectional study was conducted from December to March of years 2012–2016. A total of 813 asymptomatic school children whose antistreptolysin O test was negative were included. The mean age of the students was 10.5 ± 2.8 years with a range from 5 to 15 years old. Throat swab and blood sample were taking from each student. One hundred and four (12.8%) healthy students were found to be *Streptococcus pyogenes* carriers. Pharyngeal *Streptococcus pyogenes* carriage rate was statistically insignificant among different age groups. However, it was found to be more common among females (66, 15%) than males (38, 10%) with statistically significant difference (χ^2^ = 4.52, *P* = 0.04). This study showed a high asymptomatic carriage rate of *Streptococcus pyogenes* in the throat of healthy school children in Sana’a city, Yemen.

## Introduction

*Streptococcus pyogenes* (*S. pyogenes*) is a beta-hemolytic streptococcus which is classified as Lancefield Group A *Streptococcus* (GAS). *S. pyogenes* has remained a significant human pathogen for centuries. It causes a wide variety of infections in humans ranging from mild skin and upper respiratory tract infections to severe life-threatening conditions such as septicemia, pneumonia, necrotizing fascitis and streptococcal toxic shock syndrome. *S. pyogenes* is the most common bacterial cause of pharyngitis which is more common in settings of poverty [1].

The prevalence of streptococcal pharyngitis is highest in children older than 3 years. Nevertheless, streptococcal pharyngitis is uncommon in neonates, which may reflect a protective role of a passive immunity from mothers to newborns [2]. *S. pyogenes* has a peak incidence in children at 5–15 years of age and has been described as a ‘hazard’ in school-aged children. Global, each year, approximately 15% of school-aged children are estimated to have GAS pharyngitis. Rheumatic fever (RF) and rheumatic heart disease (RHD) are potential complications of untreated GAS pharyngitis. RHD is typically characterized by gradual damage to heart valves provoked by repeat exposures to *S. pyogenes*. The Global Burden of Disease Study estimated that more than 33 million people are living with RHD and 319,400 people die of the disease each year [3].

*Streptococcus pyogenes* frequently gets colonized in the pharynx of asymptomatic persons. The carriage rate of *S. pyogenes* is estimated to be 15–20% in asymptomatic school aged children and asymptomatic. In children from high-income countries, prevalence was 8.4–12.9% [4, 5]. While pharyngeal colonization is generally harmless for pediatric carriers, they could transmit the infection to others [6]. When screened and appropriately treated with antibiotics, pharyngeal carriers can be prevented from spreading respiratory infections in the community. This in turn would reduce the incidence of life-threatening post-infectious sequelae such as RHD which reported to be higher in Yemen than that reported from neighboring countries [7].

To our knowledge, there are no previous studies on *S. pyogenes* carriage rate among school children in Yemen. Therefore, this study was performed to investigate the prevalence of pharyngeal streptococcal carriage among healthy school children in Sana’a city, the capital of Yemen.

## Main text

### Methods

#### Study design and area

A cross-sectional study was conducted from December to March of years 2012–2016. The study was performed in winter/spring because previous studies reported seasonal variations for *S. pyogenes* infections which found to be higher in winter/spring but lower in summer/autumn [8–11]. Eight public primary schools at Sana’a city were chosen randomly by clustering sampling. These schools were Khalid Ibn Al-walleed school, Arwa school, Sokeinah school, Hamra’a Alb school, Al-Medhar school, Tarim school, Ahmad Al-Ghashmi school and Imam Ali school.

#### Study population

A total of 813 school children were enrolled in this study. Simple random sampling was used to choose participants from each school using the lists of all students in each school. Pharyngeal streptococcal carriage was defined as a positive throat culture for *S. pyogenes* without specific immune response to *S. pyogenes* [12]. Antistreptolysin O antibody (ASO) test was used as indicator for specific immune response to *S. pyogenes*.

#### Exclusion criteria

School children were excluded from the study if they had: signs or symptoms which showed upper respiratory tract infections, tonsillectomy, antibiotic usage 3 weeks before sample collection and if their ASO test was positive. Students older than 16 years old were also excluded.

#### Specimen collection and examinations

##### Throat swab

Process was explained to each student before throat swabbing. In a good light, the child was asked to open his/her mouth as wide as possible. Using a tongue depressor, the investigator looked carefully for any signs of inflammation and for presence of any exudates or pus on tonsils. If there were not any symptoms, the investigator collected throat swab by rubbing sterile cotton swab over the tonsillar area without touching the tongue or lips to minimize contamination by oral microbiota. The collected swabs were then placed in Amies transport media, labeled and delivered to the microbiology laboratory. At the laboratory, the throat swabs were inoculated on blood agar media with making stabs on blood agar to reduce oxygen levels at the stabbing areas. The inoculated blood agar plates were incubated at 35–37 °C in candle jars for 24–48 h. After incubation, plates were investigated for small gray beta hemolytic colonies particularly at the stabbing areas. Gram positive, beta-hemolytic streptococci which are catalase negative were subcultured on a new blood agar plate for bacitracin (0.04 U) sensitivity [13].

##### Venous blood

Procedure was explained to each student before blood withdrawn. Two millilitre of venous blood was collected from each student into plain vacutainer tube. Blood tubes were labeled and transported to the serology lab. In the lab, blood samples were centrifuged for 15 min at 3000 rpm and serum was separated. Sera were then tested for presence of ASO using latex agglutination test (ASO-latex, Spinreact, Spain). ASO levels equal or less than 200 IU/ml were reported as negative. All students whose ASO test was negative were enrolled in this study while those whose ASO test was positive were excluded from the study.

#### Ethical considerations

The study was approved by the Faculty of Medicine and Health Sciences, Sana’a University and heads of schools. Before samples collection, students’ parents gave a written informed consent. School children gave a verbal consent which was approved for children by the ethical committee after their parents gave a written informed consent.

#### Statistical analysis

Data analysis was done using SPSS program version 20 (SPSS Inc., Chicago, IL, USA). Descriptive measures (mean ± standard deviation) were used for continuous variables. Frequencies and percentages were used to present categorical variables. Chi-square test was used for verifying existence of associations. P values ≤ 0.05 were considered statistically significant.

### Results

Out of 813 students, 376 were males (46%) and 437 (54%) were females. The age of the study group ranged from 5 to 15 years with mean age 10.5 ± 2.8 years. Two hundred and three (25%) students were in age group 5–8 years, 379 (46.6%) students were in age group 9–12 years and 231 (28.4%) students were in age group > 12 years, Table 1.

**Table 1:** Beta hemolytic streptococci isolated from students in different age groups

Age groups	Beta-hemolytic streptococci		No pathogenic bacteria		Total		χ^2^	*P*
	No.	%	No.	%	No.	%		
5–8 years	43	21.2	160	78.8	203	25	1.11	0.58
9–12 years	70	18.5	309	81.5	379	46.6		
> 12 years	40	17.3	191	82.7	231	28.4		
Mean ± SD	10.5 ± 2.8 years							
Total	153	18.8	660	81.2	813	100		

*SD* standard deviation

Beta-hemolytic streptococci were isolated from 153 (18.8%) students. Beta hemolytic streptococci were isolated from 43 (21.2%) students in age group 5–8 years, 70 (18.5%) students in age group 9–12 years, 40 (17.3%) students in age group > 12 years with no statistically significant difference (χ^2^ = 1.11, *P* = 0.58), (Table 1). *S. pyogenes* was isolated from 104 (12.8%) students. It was isolated from 28 (14%) students in age group 5–8 years, 50 (13%) students in age group 9–12 years and 26 (11%) students in age group > 12 years with no statistically significant difference (χ^2^ = 0.73, *P* = 0.69), (Table 2). *S. pyogenes* was found to be more common among females (66, 15%) than males (38, 10%) with statistically significant difference (OR = 1.6, χ^2^ = 4.5, *P* = 0.04), (Table 3).

**Table 2:** *Streptococcus pyogenes* isolated from school children in different age groups

Age groups	*S. pyogenes*		Negative culture for *S. pyogenes*		Total		χ^2^	*P*
	No.	%	No.	%	No.	%		
5–8 years	28	14	175	86	203	25	0.73	0.69
9–12 years	50	13	329	87	379	46.6		
> 12 years	26	11	205	89	231	28.4		
Total	104	12.8	709	87.2	813	100		

χ^2^ = Chi square ≥ 3.84 is considered statistically significant

P = probability value ≤ 0.05 is considered statistically significant

**Table 3:** *Streptococcus pyogenes* isolated from male and female students

Isolated bacteria	Males		Females		Total		OR	95% CI	χ^2^	*P*
	No.	%	No.	%	No.	%				
*S. pyogenes*	38	10	66	15	104	13	1.6	1.03–2.42	4.5	0.04
Other beta-hemolytic streptococci	338	90	371	85	709	87				
Total	376	46	437	54	813	100				

### Discussion

*Streptococcus pyogenes* throat carriage is an important public health problem because a small minority of untreated streptococcal pharyngitis may trigger RF and RHD. Previous studies reported *S. pyogenes* to be isolated more often from children pharynges than in adults. Throat cultures are still the “gold standard” for isolation of *S. pyogenes* [7]. GAS carrier is defined as an individual who has a positive throat culture for *S. pyogenes* but does not develop ASO antibodies response [12]. Little is known about the *S. pyogenes* throat carriage rate among school children in Yemen. Therefore, our study is performed to evaluate the *S. pyogenes* carriage rate among school children in Yemen.

Carriage rate of β-hemolytic streptococci among school children was 18.8% while the carriage rate of *S. pyogenes* was 12.8%. The high carriage rate in our study may be attributed to the microbe spreading from one to another in overcrowded areas, poor hygiene and lack of awareness on the mode of microbial disease transmission. Carriage rate varies from high to low rate based on study reports performed in different countries. Similar high *S. pyogenes* carriage rates were reported from Argentina (14.2%), Egypt (16%), Ethiopia (9.7%), India (16.3%), Nepal (10.8%), Turkey (14.3%) and Uganda (16%) [14–20].

Regarding carriage rate of β-hemolytic streptococci and *S. pyogenes* with age of the students, the carriage rate decreased with age. However, this decrease was non-significant among different age-groups which might be attributed to a limited age range of 5–15 years. This finding is in agreement with Carapetis et al. who showed that *S. pyogenes* prevalence to decrease with age [21]. It is also in agreement with studies reported from different countries [16–19].

Our study revealed that the carriage rate of *S. pyogenes* was higher in females than males. Similar result was reported by Nayiga et al. and Vijaya et al., who isolated *S. pyogenes* more frequently from females than males [20, 22]. Although Dumre et al. found the *S. pyogenes* carriage to be slightly higher among girls, the result was statistically insignificant [23]. In contrast, other conducted studies showed that the carriage rate for boys and girls were similar [17, 18, 24]. Higher *S. pyogenes* carriage among females than males in our study could be related to the more frequent distribution of emm87 (51%) and emm28 (14%) *S. pyogenes* genotypes among Yemeni children [25]. In a Pan-European study of GAS genotype distributions, emm28 and emm87 were found to be significantly predominate in females than males while genotypes emm81 and emm83 were significantly overrepresented among males [26].

### Conclusion and recommendation

Asymptomatic carriage rate of *S. pyogenes* is high among school children. Carriage rate among females was higher than males. Further studies to exclude transient carriers by taking two swabs 3 month’s intervals is required.

## Limitations

Many students and/or their parents refused blood withdrawal for ASO test. Furthermore, bacitracin discs were unavailable for a while during study due to economic blockade on Yemen since 2015.

## Data Availability

The data that support the findings of this study are available. Anyone interested can get upon reasonable request from corresponding author.

## Abbreviations

ASO: antistreptolysin O
GAS: group A streptococci
*S. pyogenes*: *Streptococcus pyogenes*
